# Design and Manufacturing of Artificial Composite Stone Using Waste Limestone and Glass-Based Reinforcements

**DOI:** 10.3390/polym18091040

**Published:** 2026-04-24

**Authors:** Şükrü Çetinkaya

**Affiliations:** Mechanical Engineering Department, Engineering Faculty, Dicle University, Diyarbakır 21280, Türkiye; scetinkaya@dicle.edu.tr

**Keywords:** artificial composite stone, waste limestone, glass reinforcement, polyester resin, microstructure

## Abstract

Artificial composite stones have recently attracted attention as multifunctional materials for construction and defense-related applications. In this study, a novel composite stone was developed using waste limestone as the primary mineral filler, combined with an unsaturated polyester resin matrix and reinforced with glass powder and chopped glass fibers. The influence of binder content and reinforcement type on physico-mechanical and microstructural behavior was investigated. Experimental characterization included water absorption, compressive strength, abrasion resistance, acid resistance, and optical microscopy. The results demonstrated that fine fillers improved matrix densification and reduced porosity, while short glass fiber reinforcement enhanced load-bearing capacity. Abrasion resistance and durability were found to depend on binder content and particle packing characteristics. Overall, the developed composite material exhibits promising mechanical performance, low water absorption, and improved durability, suggesting its potential as a candidate material for applications requiring environmental resistance, including potential use in defense-related camouflage applications.

## 1. Introduction

Composite materials have gained a central role in modern engineering applications owing to their high specific strength and stiffness, low density, corrosion resistance, and exceptional design flexibility. By enabling the controlled combination of matrix and reinforcement constituents, polymer matrix composites allow mechanical, physical, and functional properties to be tailored within a single engineered system [1,2]. These advantages have led to their widespread adoption in aerospace, transportation, marine structures, civil infrastructure, and defense-related applications, where lightweight design and long-term durability are critical performance requirements [3,4].

Classical studies on polymer-based composites have established the fundamental role of matrix–reinforcement interactions in determining mechanical performance. Thomason [5] demonstrated that the micromechanical behavior of glass fiber-reinforced polymers is strongly governed by interfacial adhesion and fiber efficiency. Rohton [6] provided a comprehensive framework for particulate-filled polymer composites, emphasizing the influence of particle size, distribution, and filler content on stiffness and matrix continuity. Similarly, Soutis [4] highlighted the structural advantages of fiber-reinforced composites, particularly in applications requiring high strength-to-weight ratios and durability.

In recent decades, the rapid evolution of defense platforms and field-deployed systems has further intensified the demand for multifunctional materials capable of maintaining structural integrity under harsh environmental and mechanical loading conditions. Materials used in military applications must exhibit resistance to moisture ingress, abrasion, temperature fluctuations, and chemically aggressive environments while preserving mechanical reliability during prolonged outdoor exposure [7,8,9]. Consequently, polymer-based composite systems have emerged as strong alternatives to conventional metallic and monolithic stone materials in a wide range of defense- and security-related applications.

Beyond structural performance, camouflage and concealment have become increasingly important material design criteria for defense-oriented systems. Components such as protective enclosures, field structures, sensor housings, and unmanned platforms must visually integrate with natural terrains while retaining adequate mechanical strength and environmental durability. Despite the extensive literature on polymer composites, artificial or engineered stone composites specifically designed for camouflage-compatible defense applications remain relatively underexplored. Existing engineered stone systems are predominantly optimized for architectural or decorative purposes, relying on ultra-fine mineral fillers and aesthetic considerations rather than mechanical robustness and outdoor durability [6].

Sustainability considerations further strengthen the motivation for developing artificial composite stones. Mining operations and natural stone processing industries generate large quantities of mineral residues and fine sludge, the disposal of which poses significant environmental and economic challenges. The reuse of mineral waste in polymer-based composites aligns with circular economy principles and sustainable construction strategies, offering pathways for resource efficiency and waste valorization [10]. Previous studies on polymer concrete and particulate-filled polymer composites have demonstrated that the incorporation of mineral fillers can enhance matrix densification, abrasion resistance, and dimensional stability, which are essential attributes for outdoor and structural applications [11,12].

From a materials-design perspective, the performance of polymer-bonded stone composites is governed by particle packing density, filler size distribution, binder content, and interfacial bonding quality. Fine fillers and glass powders are frequently employed to reduce internal porosity and improve interfacial contact between the polymer matrix and mineral aggregates, leading to reduced water absorption and enhanced mechanical resistance [13]. Complementary to particulate optimization, fiber reinforcement represents a well-established strategy for improving load transfer and suppressing crack initiation and propagation in polymer-based composites [5,14,15].

Among available reinforcement options, glass and basalt fibers have been widely investigated due to their favorable mechanical properties and durability in outdoor environments. Reviews by Fiore et al. [16] and Sim et al. [17] highlight the effectiveness of mineral-based fibers in enhancing stiffness, strength, and environmental resistance in polymer composites. However, fiber length, dispersion quality, and compatibility with the polymer matrix remain critical parameters governing the overall performance of short-fiber-reinforced systems [18].

In parallel with mineral-filled and fiber-reinforced systems, bio-based composite materials have been explored as environmentally friendly alternatives. Comprehensive reviews on natural-fiber and bio-based composites report notable improvements in stiffness and strength through reinforcement architecture and surface treatment strategies [19,20]. Nevertheless, bio-based systems often suffer from moisture sensitivity, limited thermal stability, and reduced long-term durability, which restrict their applicability in outdoor, structural, and defense-related environments when compared with mineral-filled thermoset composites [20].

Unsaturated polyester resins remain among the most widely used thermoset matrices due to their low cost, ease of processing, good chemical resistance, and balanced mechanical performance. Their compatibility with particulate fillers and chopped fiber reinforcements makes them particularly attractive for engineered stone and construction-related composite applications [2,13]. Recent studies on unsaturated polyester composites reinforced with chopped fibers have demonstrated that matrix densification, binder fraction, and fiber dispersion critically influence compressive strength, water absorption, and durability, underscoring the relevance of optimized short-fiber architectures for outdoor applications [21]. In addition, recent studies have shown that resin modification and peroxide-curing behavior can significantly affect mechanical performance and interfacial adhesion in polymer-based systems [22,23]

Within this framework, the present study develops an artificial composite stone utilizing waste limestone particles as the primary mineral constituent, reinforced with 3.2 mm chopped glass fibers and 200 µm glass powder, and bonded using an unsaturated polyester resin matrix. Two nominal binder contents (25% and 30%) were investigated to evaluate their influence on mechanical performance, physical durability, and microstructural densification. The composite specimens were characterized through uniaxial compressive strength testing, atmospheric-pressure water absorption, wide-disc abrasion resistance, surface durability assessment under aggressive exposure conditions, and microstructural analysis using optical microscopy. The findings provide insight into the potential of mineral waste-based, fiber-reinforced artificial composite stones as sustainable and mechanically robust materials for applications requiring environmental resistance and structural reliability.

Despite the extensive body of research on polymer-based composites and engineered stones, studies specifically addressing the combined use of waste limestone, micro-scale glass powder, and short glass fiber reinforcement within a single unsaturated polyester matrix system remain limited. Furthermore, the influence of binder content on the coupled physico-mechanical and microstructural behavior of such systems has not been comprehensively evaluated, particularly in the context of durability-oriented outdoor applications.

Therefore, the novelty of this study lies in (i) the integrated use of multi-scale reinforcement (particulate and short fibers) within a waste-based composite system, (ii) the systematic comparison of different binder contents, and (iii) the combined evaluation of mechanical performance and microstructural densification. In addition, the findings provide practical insight into the development of sustainable composite materials with enhanced durability and potential applicability in demanding environmental conditions.

## 2. Materials and Methods

Artificial composite stones have been developed as advanced material systems for applications in both the construction industry and defense sector [16]. In the present study, artificial composite stone specimens were fabricated using an unsaturated polyester resin matrix combined with various reinforcements, including 200 µm glass powder, 3.2 mm chopped glass fibers, 6 mm chopped glass fibers, 6 mm chopped basalt fibers, and 12 mm chopped basalt fibers.

To establish the polymer matrix network, İLKESTER IR-100 unsaturated polyester resin (İlkester, Türkiye) was employed together with a curing system consisting of 6% cobalt octoate accelerator and BUTANOX M50 methyl ethyl ketone peroxide (MEKP) hardener (AkzoNobel, The Netherlands). This formulation enabled effective cross-linking and ensured adequate mechanical integrity and stability during and after the curing process. The fundamental physical and mechanical characteristics of the unsaturated polyester resin (UPR), which served as the matrix phase in the composite formulations, are provided in Table 1.

The physico-mechanical properties of the waste limestone used in the composite formulations are presented in Table 2. All tests were conducted in accordance with the relevant TS EN standards using calibrated laboratory equipment. Compressive strength tests were performed using a hydraulic compression testing machine with controlled loading rate, while water absorption measurements were carried out using a precision analytical balance.

**Figure 1:**
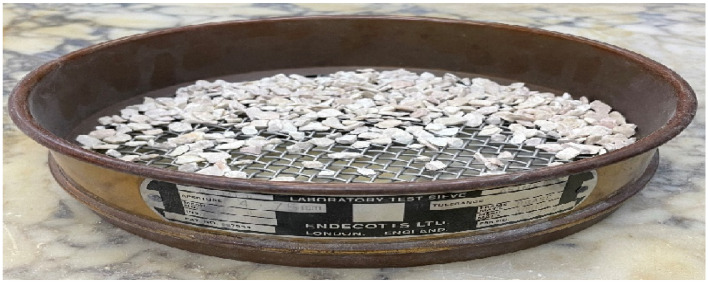
Materials retained on the 4.75 mm sieve during the preliminary screening of waste limestone.

**Figure 2:**
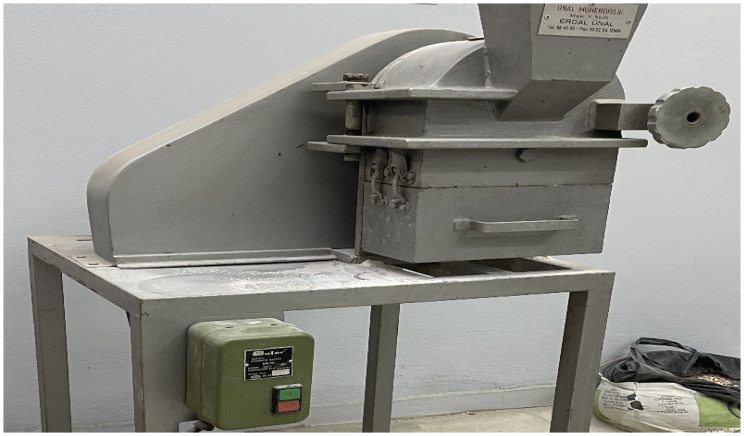
The milling device used for processing the waste limestone.

To produce the artificial composite stones, the collected waste limestone was first screened to remove undesirable impurities. The material was passed through a sieve with an aperture size of 4.75 mm, as shown in Figure 1. The retained particles on the sieve surface, representing the oversized fraction removed during this process, are illustrated in Figure 1. The milling device used for processing the waste limestone is shown in Figure 2.

**Figure 3:**
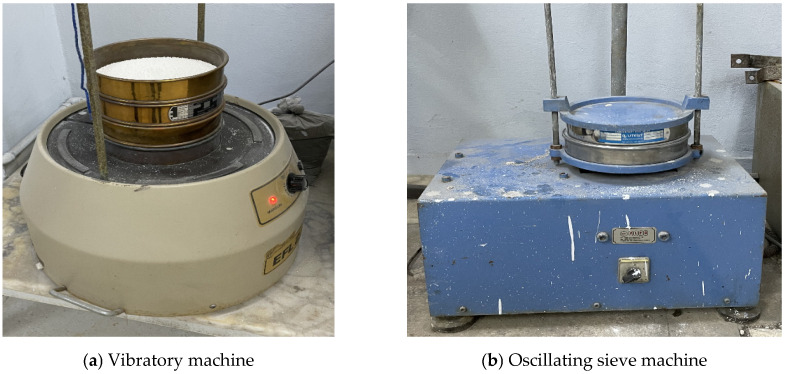
Vibratory and oscillating sieve machines used for particle size separation.

The fractured limestone particles were subsequently sieved through a series of sieves with aperture sizes of 2.00 mm, 1.19 mm, 0.707 mm, 0.500 mm, 0.300 mm, 0.212 mm, and 0.100 mm. Except for the materials retained on the 2.00 mm and 1.19 mm sieves, the fractions collected on all other sieves were separated, stored individually, and preserved for use in the composite production process. To accelerate and facilitate the sieving operation, both a vibratory sieve shaker (Figure 3a) and an oscillating sieve machine (Figure 3b) were employed.

Prior to casting the artificial composite materials, a moisture-removal procedure was applied to the crushed limestone to prevent residual humidity from creating weaknesses within the composite structure. Following the guidelines specified in the Turkish Standards Institute (TSE) document TS 699 (2009) [27]: Methods of Examination and Laboratory Testing for Natural Building Stones, each limestone fraction was subjected to a drying process. Accordingly, the crushed limestone samples were placed in a laboratory oven at a temperature of 70 ± 5 °C for a duration of 24 ± 2 h to ensure complete moisture elimination and to achieve standardized pre-conditioning.

The first mixture did not contain any reinforcing materials and was prepared solely with the resin–limestone matrix components (Table 3).

It should be noted that the “nominal binder content” values given in Table 3 represent the combined fraction of unsaturated polyester resin and cobalt accelerator. The cobalt accelerator corresponds to approximately 0.1 wt.% of the resin fraction. Therefore, the actual resin content is marginally lower than the nominal binder content; however, this difference (<0.1%) does not significantly affect the comparative evaluation between the 25% and 30% binder systems.

The selected binder contents (25% and 30%) are higher than those commonly reported in conventional polymer concrete systems, where binder contents typically range between 8% and 15%. However, in the present study, higher resin fractions were intentionally employed to ensure adequate coating of fine limestone particles and glass-based reinforcements, as well as to enhance matrix continuity and reduce internal porosity. The use of multi-scale fillers, including micro-sized glass powder and fine limestone fractions, increases the total surface area of the solid phase, thereby requiring a higher binder content to achieve proper wetting and homogeneous dispersion. In addition, one of the primary objectives of this study was to achieve low water absorption and improved environmental durability, which are strongly influenced by matrix densification. Therefore, the selected binder contents were not intended to represent optimized industrial formulations, but rather to enable a systematic comparative evaluation of the influence of binder fraction on the physico-mechanical performance of the composite system.

The compositional details of all mixtures are presented in Table 3. The formulations were designed to systematically investigate the effect of reinforcement type while maintaining a consistent limestone particle size distribution across all mixtures. Mixtures 1 and 2 were prepared without reinforcement and serve as reference compositions. Mixtures 3 and 4 include 200 µm glass powder as a micro-scale filler, whereas Mixtures 5 and 6 incorporate 3.2 mm chopped glass fibers to evaluate the effect of short fiber reinforcement.

All materials were weighed using a high-precision analytical balance (accuracy: 0.0001 g). The polymer mixture required for casting the artificial composite stones was also prepared by weighing all components using a high-precision analytical balance. An accelerator containing 6% cobalt was added to the unsaturated polyester resin. The resin–cobalt system was then thoroughly mixed to ensure complete homogenization. Subsequently, the pre-measured limestone powders and particles were incorporated into the mixture and blended until a uniform consistency was achieved. MEK-P was added as the final component. Following the addition of MEK-P, the gelation time of the composite mixture ranged from approximately 8 to 12 min; therefore, any material that could not be cast within this period became unusable.

**Figure 4:**
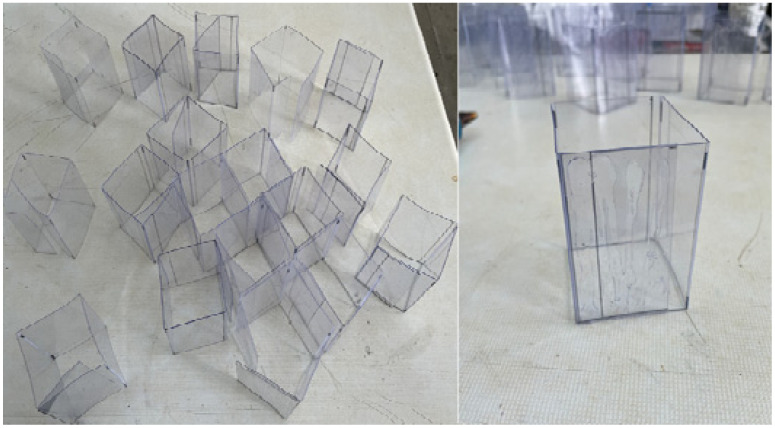
Folded acetate sheets and the bonded corner using hot silicone.

For mold preparation, 500-micron-thick acetate sheets were cut into 70 mm × 250 mm sections. Acetate was selected as the mold material due to its suitability and ease of shaping. According to the TS 699 standard, cubic specimens with dimensions of 50 mm × 50 mm × 50 mm are required for atmospheric-pressure water absorption tests and uniaxial compressive strength tests. To obtain these specimens, the necessary measurements were marked on the acetate sheets and subsequently cut to the required dimensions. The acetate sheets were folded along the marked measurement lines, and the folded edges were bonded using a hot silicone gun to form a rectangular prism-shaped mold. This procedure ensured dimensional stability and leak-proof joints during the casting of the composite mixtures (Figure 4).

After vacuum treatment, the specimens were left to cure under ambient laboratory conditions (23 ± 2 °C) for 24 h before demolding.

After demolding, all specimens were stored under controlled laboratory conditions (23 ± 2 °C and relative humidity of 50 ± 5%) for an additional 7 days to allow completion of post-curing reactions of the unsaturated polyester matrix. No elevated-temperature post-curing was applied.

All mechanical and physical tests were conducted on the 8th day after casting. Prior to each specific test, specimens were conditioned in accordance with the respective TS EN standard requirements (e.g., oven drying at 70 ± 5 °C for 24 ± 2 h for water absorption and compressive strength tests). This standardized curing and conditioning protocol ensured consistency and reproducibility across all experimental measurements. It should be noted that the oven drying applied prior to testing was performed solely for specimen conditioning in accordance with TS EN standards and is not a post-curing process. This procedure was not intended to enhance polymer crosslinking or alter the degree of polymerization of the resin matrix.

**Figure 5:**
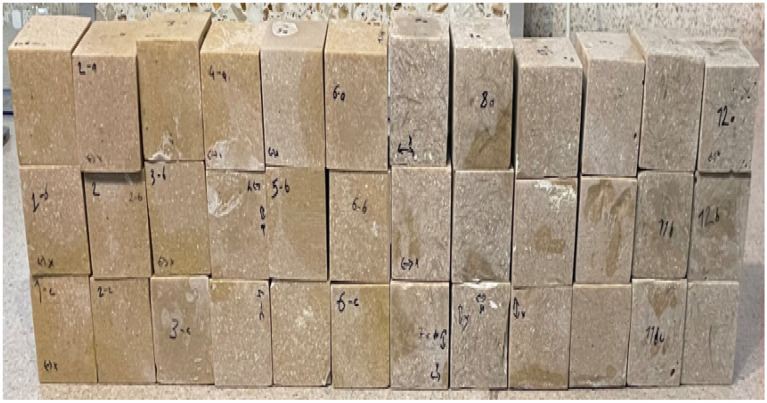
Composite specimens after demolding.

A selection of the cured composite specimens removed from their molds is shown in Figure 5.

The specimens removed from the molds were subsequently prepared for optical microscopy (OM) analysis. To obtain a smooth and observable surface, one face of each specimen was ground using a metallurgical grinding machine equipped with abrasive papers of progressively finer grit sizes, including 80, 100, 120, 240, 300, 480, 600, 800, 1000, 1200, and 1500 grit.

## 3. Experimental Studies

### 3.1. Water Absorption Test Under Atmospheric Pressure

**Figure 6:**
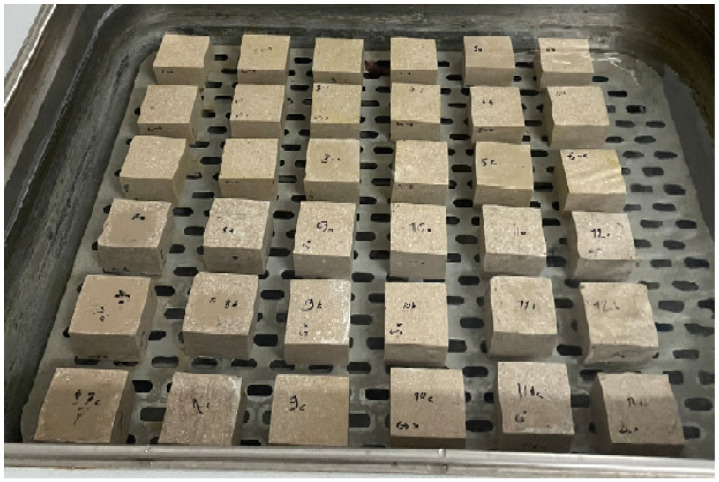
Water absorption tank used for atmospheric pressure water absorption testing in accordance with TS EN 13755.

According to the TSE TS 699 standard, water absorption under atmospheric pressure must be performed in accordance with TS EN 13755 [28]. The TS EN 13755 specification states that cubic specimens with dimensions of 50 ± 5 mm are required for this test. It also specifies that the specimens must be dried in a laboratory oven at 70 ± 5 °C for 24 ± 2 h prior to testing. After oven-drying, the specimens are weighed using an analytical balance with a precision of 0.0001 g, and their dry masses are recorded. The water temperature in the absorption tank (Figure 6) must be maintained at 20 ± 10 °C as required by the standard. The dried specimens are placed into the tank, and the water level is gradually raised over time according to the prescribed procedure. After 48 ± 2 h of immersion, the specimens are removed from the water, lightly wiped with a damp cloth, and weighed within one minute. The recorded mass is noted, and the specimens are returned to the water. This process is repeated every 24 ± 2 h. The test is considered complete when the difference between two consecutive mass measurements does not exceed 0.1%. The water absorption value under atmospheric pressure is calculated using Equation (1):

(1) $$A_{b}=\frac{m_{s}-m_{d}}{m_{d}}\times 100$$

where: $m_{s}$, mass of the saturated specimen (g), $m_{d}$, mass of the dry specimen (g), $A_{b}$, water absorption under atmospheric pressure (%).

### 3.2. Determination of Surface Appearance Change Under Atmospheric Conditions

This test, described in the TS 699 standard, was conducted as follows:

The surfaces of the specimens were ground and polished, after which they were exposed to a 1% hydrochloric acid solution until visible alterations appeared. The specimens were immersed in the solution for one day, then removed, rinsed thoroughly, and the changes observed on their surfaces were recorded.

### 3.3. Uniaxial Compressive Strength Test

**Figure 7:**
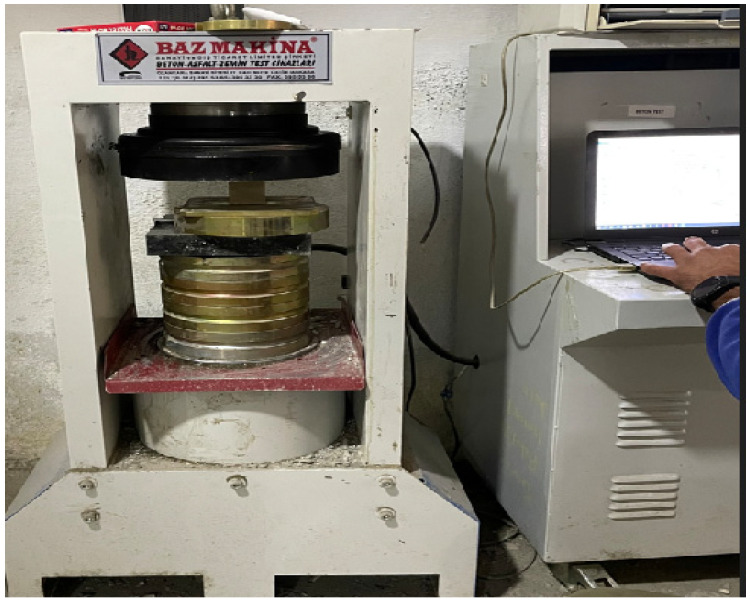
Uniaxial compressive strength test machine.

This test is required by TS 699 to be performed in accordance with the TS EN 1926 standard. TS EN 1926 specifies that the specimens used in the uniaxial compressive strength test must be cubic with dimensions of 50 ± 5 mm. Prior to testing, the specimens must be dried in an oven at 70 ± 5 °C for 24 ± 2 h. The standard also requires that the testing machine apply load at a rate of 1 ± 0.5 MPa per second. The machine used in this study, shown in Figure 7, operates with a loading rate of 0.5 MPa per second.

**Figure 8:**
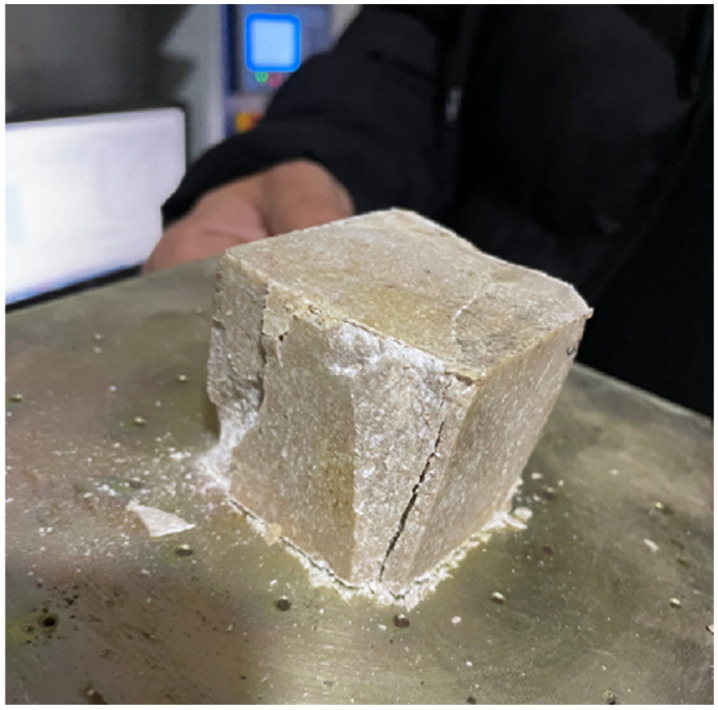
Condition of the specimen after the compressive strength test.

After measuring the specimen dimensions, the test is initiated. Once the specimen fails, the maximum load is recorded (Figure 8). The uniaxial compressive strength (R) is then calculated using Equation (2) by dividing the maximum load by the previously measured cross-sectional area of the specimen:

(2) R=F/A

where *R* uniaxial compressive strength (MPa), *F* maximum force applied to the specimen (kN), *A* cross-sectional area of the specimen (mm^2^).

### 3.4. Wide-Disc Abrasion Test

**Figure 9:**
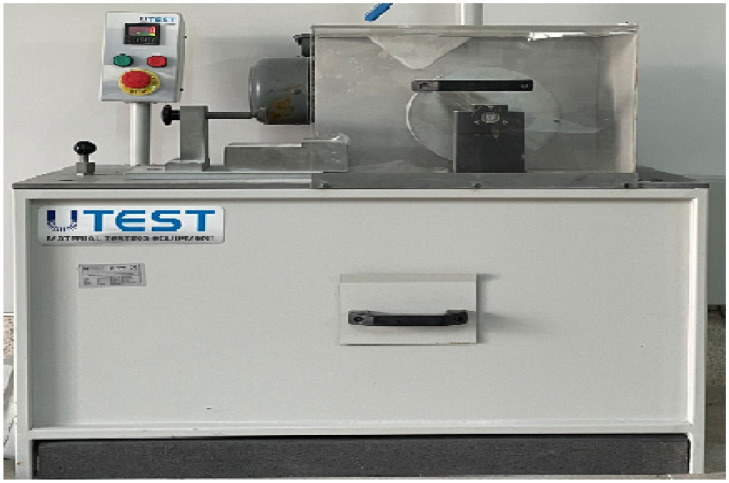
Wide-disc abrasion testing device.

This test was carried out according to TS EN 14157 [29]. The wide-disc abrasion testing device used for the experiment is shown in Figure 9.

According to the standard, rectangular specimens measuring 100 mm × 70 mm are prepared. Prior to testing, the specimens are oven-dried as in previous procedures. The abrasion device must be capable of rotating the wide abrasive disc at 75 revolutions within 60 s. Alumina is used as the abrasive medium.

During testing, the specimen is attached to the holding mechanism, where a counterweight ensures constant contact between the specimen and the rotating abrasive disc. The valve controlling the alumina powder is opened, allowing the abrasive particles to fall onto the disc. When the device is activated, the disc completes 75 revolutions in 60 s.

After the test, the abrasion marks on the specimen are traced with a marker, and wear widths are measured at three positions using a caliper with a precision of 0.05 mm to determine the extent of material loss.

### 3.5. Optical Microscopy (OM) Analysis

**Figure 10:**
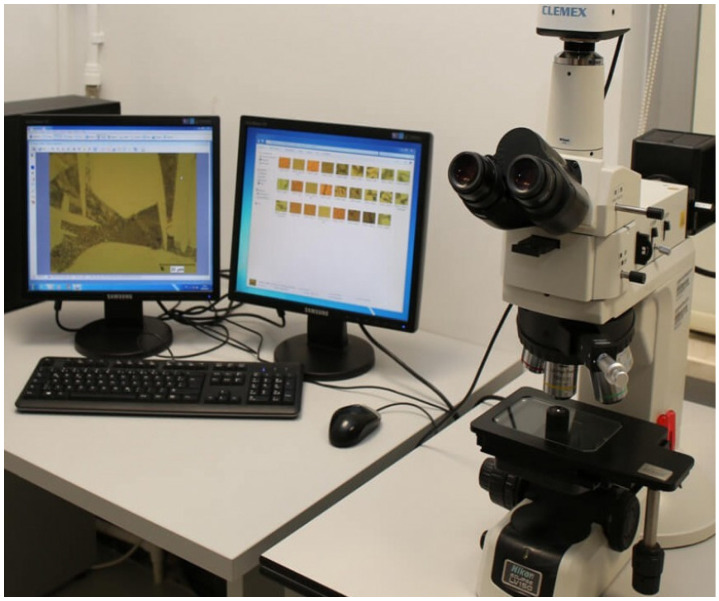
Optical microscope used for surface imaging of the specimens.

Surface examinations were performed using the optical microscope (OM) available in the Materials Laboratory at Dicle University. Specimens with one polished surface were imaged using objective lenses providing 5×, 10×, 20×, and 50× optical magnification. All captured images were recorded for subsequent analysis. The optical microscope used in the study is shown in Figure 10.

## 4. Experimental Results

### 4.1. Water Absorption Test Results Under Atmospheric Pressure

The water absorption tests were conducted in accordance with the TS EN 13755 standard. The results showed that some specimens exhibited water absorption values reaching up to 0.1% under atmospheric pressure, although considerable variability was observed among the mixtures. For each mixture, three specimens were tested, and the average absorption values were calculated. Table 4 presents the mean atmospheric-pressure water absorption percentages for all mixtures. The reported values for average water absorption represent arithmetic mean values obtained from three replicate specimens (*n* = 3). Due to the limited number of replicates, standard deviation values and statistical significance analysis were not included. The results should therefore be interpreted as comparative trends rather than statistically validated differences. Nevertheless, all measurements were conducted under controlled laboratory conditions using consistent procedures to ensure repeatability and minimize experimental variability. Future studies will focus on increasing the number of specimens and incorporating detailed statistical analysis to improve the robustness of the results.

In several cases, the specimens exhibited no measurable mass change even after 72 h of immersion, with some samples showing a difference of less than 0.01 g on the analytical balance.

According to TSE EN standards, the water absorption values of traditional natural stones typically range between 0.1% and 2%, while engineered stone materials generally exhibit values below 0.1%. In the present study, all mixtures demonstrated significantly lower water absorption values, with the minimum value reaching approximately 0.005%, indicating a highly dense and impermeable microstructure.

The water absorption results of the first six mixtures reveal distinct variations in performance despite all mixtures exhibiting very low absorption values (<0.04%), consistent with the hydrophobic behavior expected from polyester resin-based composite systems. The first mixture, which contains no reinforcement, shows the highest absorption within this subset (0.04%), suggesting that the absence of microfillers or fibers may permit the formation of small internal voids or less efficient particle packing. In contrast, the second mixture, despite having a reduced resin content, demonstrates a lower absorption value (0.021%), indicating that improved particle-size distribution or reduced void fraction may have compensated for the lower matrix volume. The third mixture exhibits the lowest absorption among all mixtures (0.005%), which can be attributed to the incorporation of 200 µm glass powder. The fine glass particles likely enhance microstructural densification by filling voids and improving the interfacial bonding between the matrix and mineral components, thereby inhibiting water ingress. The fourth mixture, formulated with the same reinforcement but reduced resin content, shows a moderate increase in absorption (0.026%), supporting the interpretation that insufficient resin may weaken matrix continuity and enhance capillary pathways. The fifth and sixth mixtures, both reinforced with 3.2 mm chopped glass fibers, show intermediate absorption values (0.02% and 0.03%, respectively). These results suggest that while glass fibers improve structural integrity, their relatively large size compared to microfillers may introduce localized microvoids around the fiber–matrix interface if the wetting is not optimal. Overall, the first six mixtures highlight the critical role of resin content, reinforcement size, and particle packing density in governing the water absorption characteristics of artificial composite stones.

It should also be noted that the minor variations observed between mixtures may be attributed to inherent microstructural heterogeneities within the composite system. In particulate and hybrid composites, local differences in particle packing density, size distribution, and spatial arrangement can lead to non-uniform porosity and localized variations in permeability. Regions with suboptimal packing may contain microvoids or weakly bonded interfaces, which can influence water ingress behavior. In addition, the interaction between the polymer matrix and reinforcement phases plays a significant role in governing variability. Incomplete wetting of particles or fibers, as well as variations in interfacial adhesion, may result in localized discontinuities affecting fluid transport behavior. Furthermore, slight differences in resin distribution during mixing and casting may contribute to heterogeneity in matrix continuity, particularly in systems with high filler content and multi-scale reinforcement. These factors are intrinsic to particulate composite systems and are consistent with the microstructural features observed in the optical microscopy analysis. Therefore, the observed variations should be interpreted as inherent material behavior rather than solely experimental inconsistency.

### 4.2. Results of the Surface Appearance Change Test Under Atmospheric Conditions

After removal from the hydrochloric acid solution and subsequent rinsing, the specimens were examined visually and photographed. The images revealed that the regions highlighted with yellow circles correspond to areas where the hydrochloric acid dissolved the limestone particles embedded within the artificial composite. This behavior is consistent with the well-known vulnerability of limestone to acidic environments. The pores visible within the circled regions represent voids left behind after the dissolution of the limestone.

Although the degradation behavior under hydrochloric acid exposure was clearly observed through visual inspection and surface analysis, no quantitative measurements such as mass loss percentage or post-exposure compressive strength reduction were performed in this study. Therefore, the evaluation of acid resistance is limited to qualitative assessment. Future studies will focus on quantifying degradation through mass loss measurements and mechanical property evaluation after chemical exposure.

**Figure 11:**
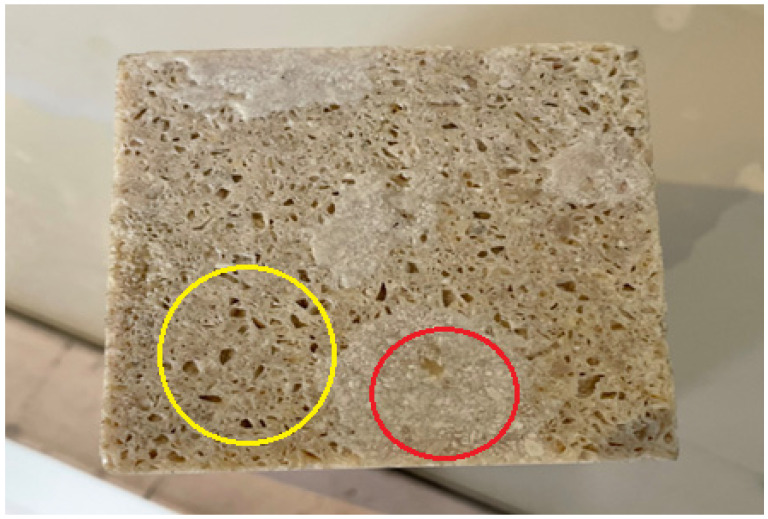
Effect of hydrochloric acid on the artificial composite stone (yellow circle indicates regions where limestone particles were dissolved by acid exposure, while red circle indicates areas where the polyester resin matrix remained intact).

In contrast, the areas indicated by red circles show regions where the unsaturated polyester resin encapsulating the limestone particles remained intact, exhibiting no visible damage. This observation reflects the high chemical resistance of unsaturated polyester resin against acidic solutions. By forming a continuous protective matrix around the limestone particles, the polyester resin appears to inhibit direct acid penetration, thereby reducing the extent of degradation within the composite. The resulting damage pattern observed after the test is presented in Figure 11.

### 4.3. Uniaxial Compressive Strength Test Results

**Figure 12:**
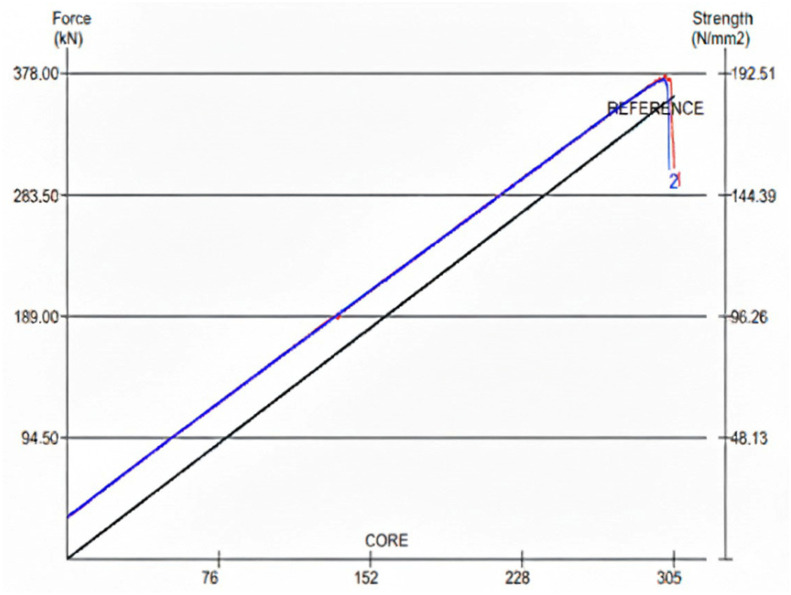
Computer-generated output of the uniaxial compressive strength test for mixture 4 (blue line represents the measured response, black line indicates the reference linear behavior, and red markings indicate the failure point).

The uniaxial compressive strength tests were performed by loading each specimen until failure. During testing, the specimen dimensions were entered into the software connected to the testing system, allowing the compressive strength values to be calculated directly in MPa. Two representative outputs obtained from the testing software are shown in Figure 12. The graphical representations of the recorded load–displacement data for all mixtures are provided in the subsequent figures.

The compressive strength results were evaluated in comparison with typical values reported for natural stones and polymer-based composite materials. According to the TSE EN, the compressive strength of natural stones varies significantly depending on their composition, generally ranging between 20 and 200 MPa, while polymer concrete and engineered stone materials often exhibit values within a similar or slightly lower range depending on binder content and reinforcement type.

In this study, the developed composite materials demonstrated compressive strength values within the lower to mid-range of this spectrum. The incorporation of chopped glass fibers contributed to improved load-bearing capacity, likely due to enhanced stress transfer and crack-bridging mechanisms within the composite structure.

Furthermore, mixtures containing micro-scale glass powder exhibited improved matrix densification, which is known to reduce internal defects and enhance compressive performance. These results indicate that both binder content and reinforcement type play a critical role in determining the mechanical performance of artificial composite stones.

The average compressive strength values were calculated from the computer-generated test results, and these averages were used to construct the graphs presented below. The results indicate that the compressive strengths of the artificial composite stones vary depending on the type of reinforcement and the resin content.

**Figure 13:**
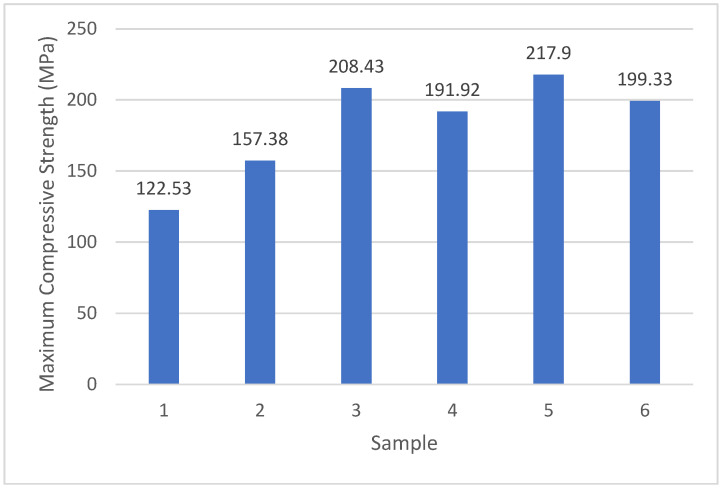
Average uniaxial compressive strength values of the composite specimens.

For mixtures reinforced with 200 µm glass powder (Mixtures 3 and 4), the specimen containing 30% resin (Mixture 3) exhibited a higher compressive strength compared with the specimen containing 25% resin (Mixture 4). This suggests that the increased resin content improves matrix continuity and enhances load transfer across the composite interface. The average compressive strength values for all mixtures are illustrated in Figure 13.

### 4.4. Disc Abrasion Test Results

**Figure 14:**
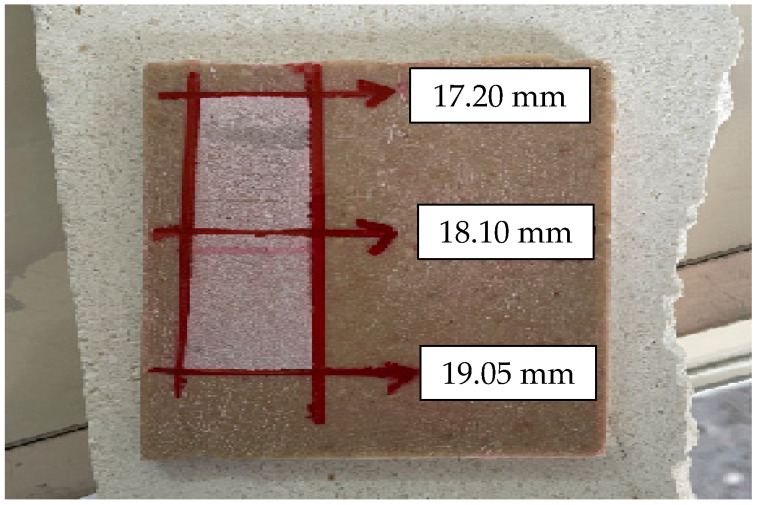
Result of the wide-disc abrasion test showing the marked wear track.

The wide-disc abrasion tests were conducted according to the TS EN 14157 standard. After abrasion, the worn regions on the specimen surfaces were outlined using a pencil and a ruler as specified in the procedure. An example of the marked abrasion pattern is shown in Figure 14.

The average abrasion widths obtained from the measurements conducted at three different points on each specimen are presented in Table 5. These values represent the mean abrasion resistance performance of each composite formulation.

The abrasion results for the first six specimens reveal noticeable variation in wear resistance among the different composite formulations. Specimen 2 exhibits the lowest wear value (15.75 mm), indicating superior abrasion resistance, whereas Specimen 1 shows the highest wear (18.10 mm), suggesting a relatively weaker microstructural integrity against abrasive loading. The improved performance of Specimens 2, 3, and 4 (with values between 15.75 and 16.70 mm) may be attributed to a more favorable balance between resin content and particle packing density, which enhances the cohesion of the composite matrix and reduces material removal during abrasion. In particular, mixtures incorporating micro-sized fillers such as fine limestone powder or 200-µm glass powder are likely to exhibit improved matrix densification, reducing pore connectivity and thereby limiting abrasive penetration depth. In contrast, higher wear values observed in Specimens 1 and 5 suggest that inadequate filler compaction or suboptimal resin distribution may have resulted in locally weaker zones susceptible to micro-fracture and material detachment under disc abrasion. Overall, the first six mixtures demonstrate that abrasion resistance is highly sensitive to filler particle size distribution, reinforcement type, and resin–aggregate interaction, consistent with the expected behavior of particulate polymer composites under sliding wear conditions.

### 4.5. Optical Microscopic Examination of Specimen Surfaces

**Figure 15:**
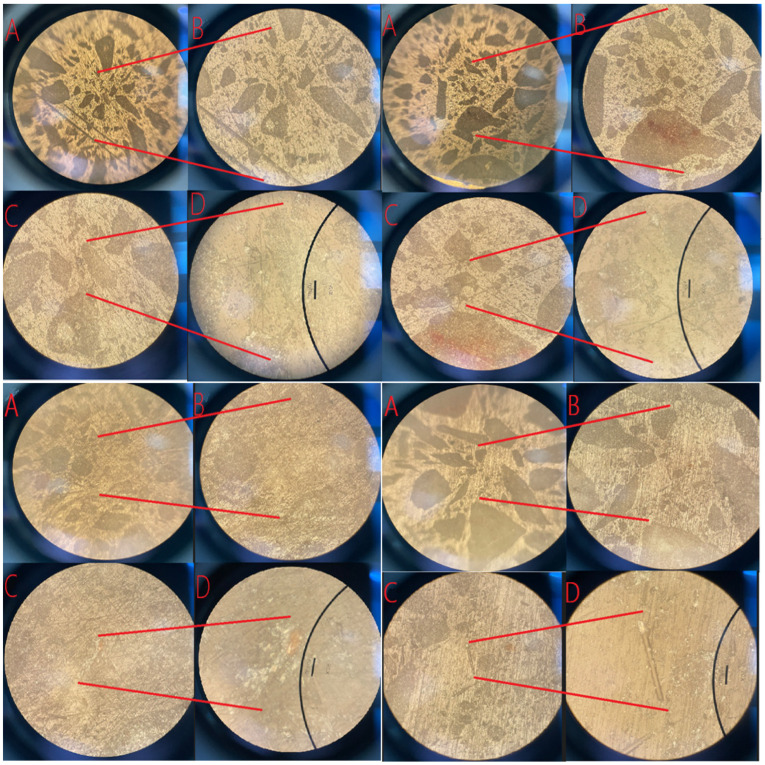
Optical microscopy images of mixtures 1, 2, 3 and 5. (**A**–**D**) correspond to magnifications of 5×, 10×, 20×, and 50×, respectively. Red lines indicate regions of interest related to surface features and particle–matrix interactions, while black lines highlight particle boundaries and interface characteristics.

Following the completion of the grinding procedure, the polished surfaces of the specimens were examined using an optical microscope (OM). The imaging process was initially performed using a 5× objective lens, after which the same regions were imaged at higher magnifications of 10×, 20×, and 50×. In Figure 15, the images labeled as “A” correspond to those captured at 5× magnification, while “B,” “C,” and “D” represent the same surface locations imaged at 10×, 20×, and 50× magnification, respectively.

In the obtained micrographs, the dark brown regions correspond to limestone particles of various sizes incorporated into the artificial composite stone. These particles are clearly distinguishable from the surrounding polymer matrix due to their contrasting optical characteristics. The progressive magnification levels enable detailed visualization of particle–matrix interfaces, surface morphology, and microstructural features such as particle distribution and potential void formations.

The optical microscopy images presented in Figure 15 reveal distinct microstructural characteristics of Mixtures 1, 2, 3, and 5 at magnifications of 5×, 10×, 20×, and 50×. Across all mixtures, the limestone particles appear as dark brown regions embedded within the lighter polyester resin matrix, reflecting the inherent contrast between mineral fillers and polymeric phases. In Mixtures 1 and 2, the particle distribution appears relatively uniform, though occasional interparticle gaps suggest the presence of minor voids or incomplete resin wetting, which is expected in particulate composites with varying granulometry. At higher magnifications (20× and 50×), the edges of the limestone particles become more clearly defined, revealing smooth dissolution boundaries and confirming the brittle nature of the mineral filler compared to the ductile resin matrix.

For Mixture 3, which contains 200 µm glass powder reinforcement, the micrographs show a noticeably denser and more homogeneously packed structure. Fine glass particles appear to occupy void spaces between limestone grains, enhancing matrix continuity and likely contributing to the improved compressive strength and reduced water absorption observed in earlier tests. The interfaces show fewer dark void regions, indicating stronger particle–matrix adhesion.

Mixture 5, reinforced with 3.2 mm chopped glass fibers, exhibits a more complex microstructure. Although the fibers are not fully visible within the field of view due to their larger aspect ratio, their presence influences the surrounding matrix morphology. The images show elongated zones of resin alignment and localized densification near fiber–matrix interfaces, consistent with fiber-induced stress redistribution within the composite. However, occasional microvoids are also present, likely originating from incomplete wetting around fiber surfaces—a common phenomenon in fiber-reinforced polyester composites.

Overall, the micrographs confirm that reinforcement type and particle-size distribution play a major role in controlling the microstructural integrity of the artificial composite stones. Mixtures incorporating fine fillers (e.g., Mixture 3) demonstrate superior densification and fewer voids, whereas mixtures with coarser particles or fiber reinforcement exhibit more heterogeneous interfaces.

## 5. Conclusions

This study developed artificial composite stones based on waste limestone and unsaturated polyester resin, reinforced with either 200 µm glass powder or 3.2 mm chopped glass fibers. The results demonstrate that both reinforcement type and nominal binder content play a decisive role in controlling matrix densification, mechanical strength, and surface durability.

It should be noted that the present study focuses primarily on the mechanical performance and environmental durability of the developed composite materials. While the findings indicate promising characteristics for applications requiring environmental resistance, no specific experimental validation related to camouflage performance (e.g., optical reflectance, thermal signature, or radar absorption) was conducted within the scope of this work. Therefore, such applications should be considered as potential directions for future research.

All mixtures exhibited very low water absorption values below 0.04%, with the minimum value reaching approximately 0.005% in glass powder–reinforced specimens, indicating a highly dense and impermeable microstructure. The incorporation of 200 µm glass powder significantly improved particle packing efficiency and reduced internal porosity. In contrast, fiber-reinforced mixtures showed higher compressive strength due to enhanced load transfer mechanisms within the polymer matrix. However, the abrasion results indicated that increased compressive strength does not necessarily correspond to improved wear resistance, highlighting a formulation-dependent trade-off between strength and surface durability.

Acid exposure tests confirmed the susceptibility of limestone particles to aggressive environments, while also demonstrating that the polyester matrix provides partial encapsulation and contributes to improved chemical resistance compared to unbound materials. Optical microscopy observations supported these findings by revealing differences in particle packing, interfacial bonding, and void distribution among the mixtures.

Although the study provides a comparative evaluation of formulation-dependent performance, it does not include statistical population analysis or long-term durability assessment under cyclic environmental conditions. Therefore, future studies should focus on quantitative microstructural characterization, statistical validation, and long-term environmental durability to further optimize the composite system.

Overall, the developed artificial composite materials exhibit low water absorption, improved mechanical performance, and enhanced environmental resistance, indicating their potential applicability in engineering applications requiring durability and structural reliability.

## Figures and Tables

**Table 1:** Physicochemical properties of the unsaturated polyester resin in the liquid state.

Analysis Type	Unit	Test Method	Value
Density (20 °C)	g/cm^3^	ISO 1675 [24]	1.10–1.12
Viscosity (20 °C)	Cps	ISO 2555 [25]	750–900
Acid Number	mg KOH/g	ISO 2114 [26]	11–20
Gel Time	Min	ILKALEM TL-06	8–14

**Table 2:** Physico-mechanical properties of the limestone.

Test	Unit	Condition	Limestone
Compressive strength	MPa	Dry	17.8 ± 1.69
Compressive strength	MPa	Saturated	14.65 ± 2.12
Water absorption (by wt.)	%	—	8.75 ± 2.45
Density	g/cm^3^	—	2.87

**Table 3:** Composition of all composite mixtures (wt.%).

Material	Mixture 1	Mixture 2	Mixture 3	Mixture 4	Mixture 5	Mixture 6
Nominal binder content (%)	30.00	25.00	30.00	25.00	30.00	25.00
MEKP hardener (%)	0.30	0.25	0.30	0.25	0.30	0.25
Limestone (>100 µm) (%)	15.00	15.84	14.16	15.00	14.95	15.79
Limestone (<100 µm) (%)	5.00	5.84	4.16	5.00	4.95	5.79
Limestone (<200 µm) (%)	5.10	5.94	4.26	5.10	5.05	5.89
Limestone (<300 µm) (%)	7.40	8.24	6.56	7.40	7.35	8.19
Limestone (<500 µm) (%)	13.20	14.04	12.36	13.20	13.15	13.99
Limestone (<707 µm) (%)	24.00	24.84	23.16	24.00	23.95	24.79
Glass powder (200 µm) (%)	—	—	5.05	5.05	—	—
Chopped glass fiber (3.2 mm) (%)	—	—	—	—	0.30	0.30

**Table 4:** Average water absorption values under atmospheric pressure.

Mixture No.	Average Water Absorption (%)
1	0.040
2	0.021
3	0.005
4	0.026
5	0.020
6	0.030

**Table 5:** Average abrasion values from the wide-disc abrasion test.

Specimen No.	Average Abrasion (mm)
1	18.10
2	15.75
3	16.70
4	16.20
5	17.65
6	16.45

## Data Availability

The raw data supporting the conclusions of this article will be made available by the author on request.
